# Injurious Effects from Chloroform

**Published:** 1849-07

**Authors:** 


### INJURIOUS EFFECTS FROM CHLOROFORM.

It is stated in the New York Express, that Mrs. Rehlsant was put under the influence of chloroform, for the purpose of having a tooth extracted. After inhaling the article, she lay lifeless for several hours, and was finally conveyed home in a state of insensibility; ultimately she recovered her mental faculties, but has since been laboring under prostration, paralysis of the tongue, and loss of voice.

A young man in New Bedford, who inhaled chloroform for amusement, is said in the papers to have been thrown into convulsions, which lasted for sixteen hours without intermission.

In Baltimore, a medical student came near losing his life from the same cause; after inhaling chloroform for a time, he became insensible, in which state he remained for one hour and a half.

An apothecary's apprentice in Philadelphia, was thrown into convulsions by the inhalation of chloroform.

A lady of Cincinnati, in the enjoyment of good health, who inhaled chloroform, preparatory to having a tooth extracted, suddenly sank under its effects, and did not revive.

A man in New York suddenly expired, after inhaling chloroform to produce insensibility to the pain of an operation for fistula in ano.

Deaths from inhaling this agent are beginning to be recorded in the foreign journals; among those lately received by us, we find two mentioned. A girl, fifteen years of age, was troubled with onychia maligna, and it was deemed advisable by the surgeon in attendance to remove the nail. Previous to commencing the operation, she was induced to breathe the chloroform; she was speedily brought under its influence, when the operation was performed. Immediately after the removal of the nail, her face was observed to be blanched, and features altered; cold water was dashed into the face, and brandy given, but to no purpose, for she was dead. A *second* case is recorded as having happened to an apothecary's boy. He had been in the habit of breathing it for its pleasurable effects, and on the day of his death, after inhaling it for a short time, he fell forward on the counter, his face dropping into the towel on which had been poured the chloroform. On being raised he was found lifeless.

